# Supplementary figures and images for: Molecular serotyping of Haemophilus parasuis isolated from diseased pigs and the relationship between serovars and pathological patterns in Taiwan
Source: PeerJ. 2018 Nov 29;6:e6017. doi: 10.7717/peerj.6017 (PMC6275120; doi:10.7717/peerj.6017)

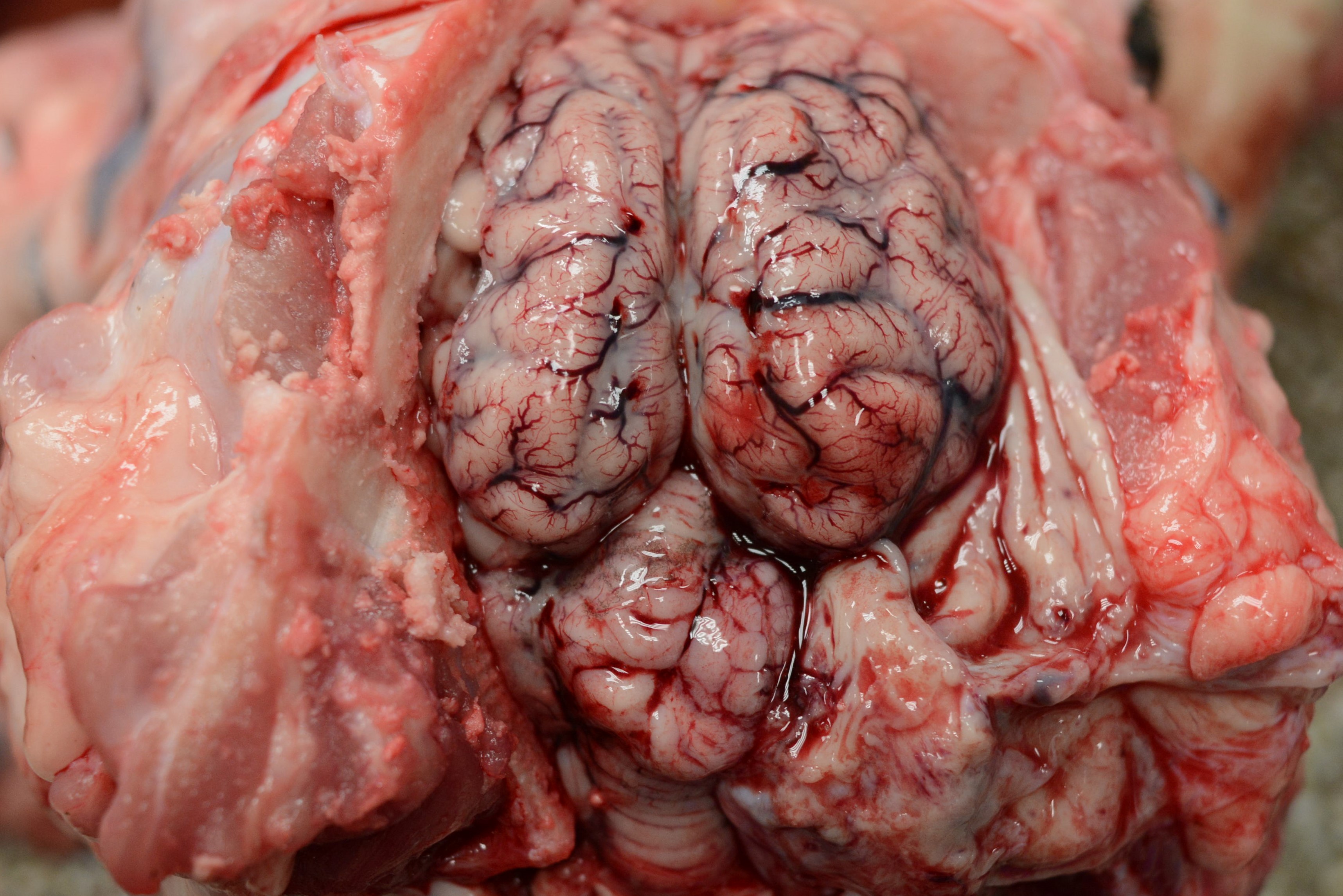

Supplement: Supplemental Information 1 [file peerj-06-6017-s001.jpg]

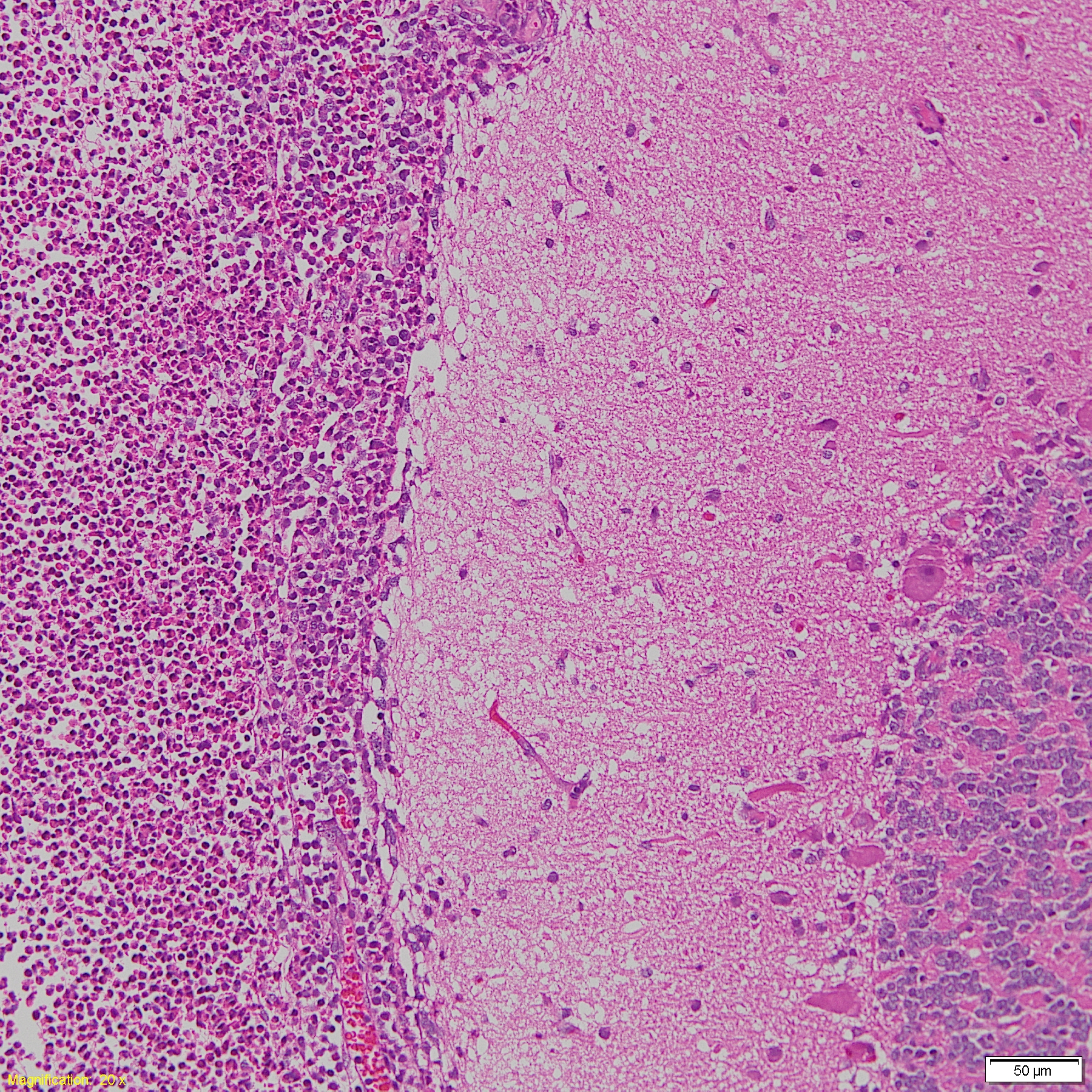

Supplement: Supplemental Information 2 [file peerj-06-6017-s002.jpg]

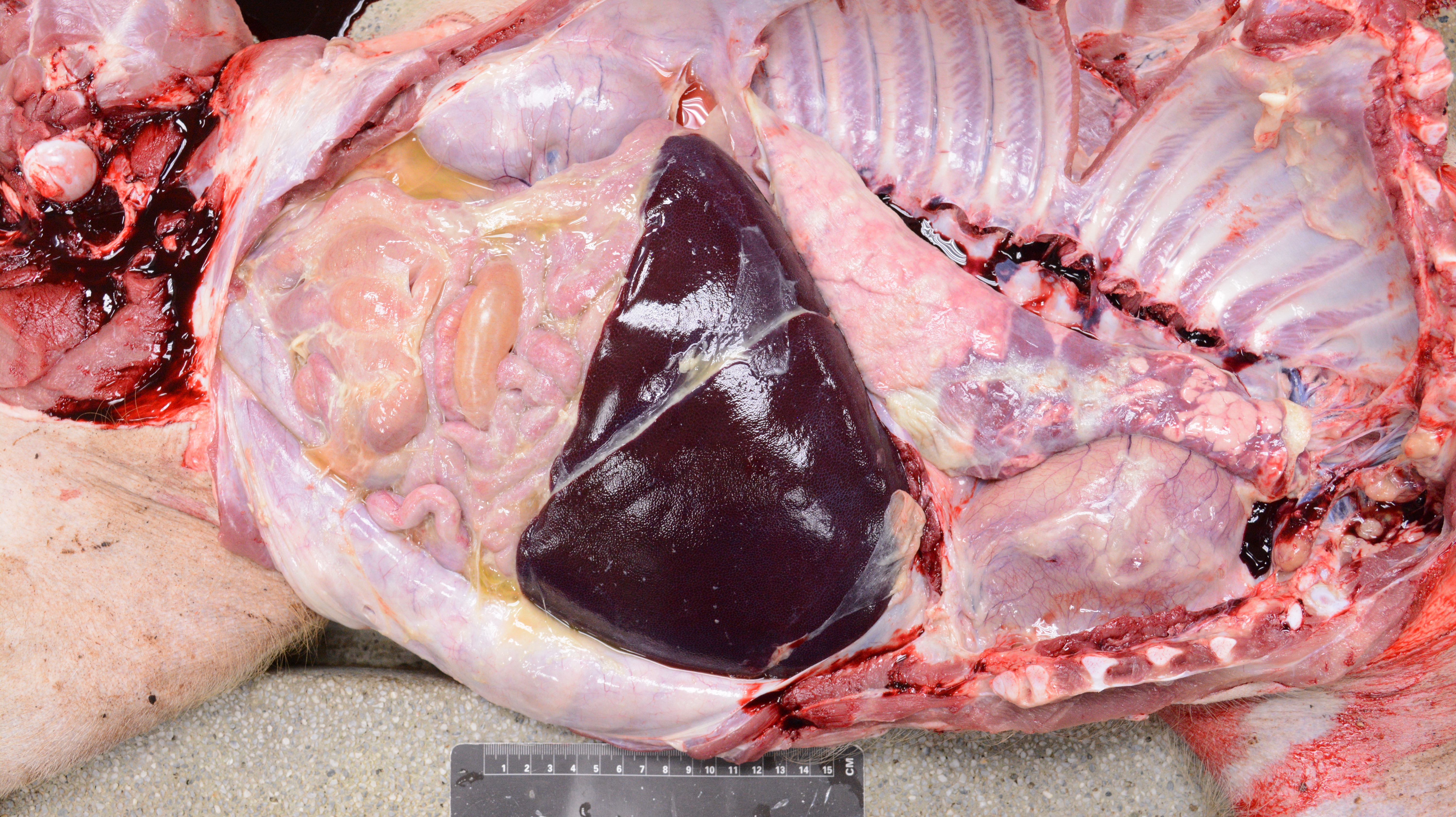

Supplement: Supplemental Information 3 [file peerj-06-6017-s003.jpg]

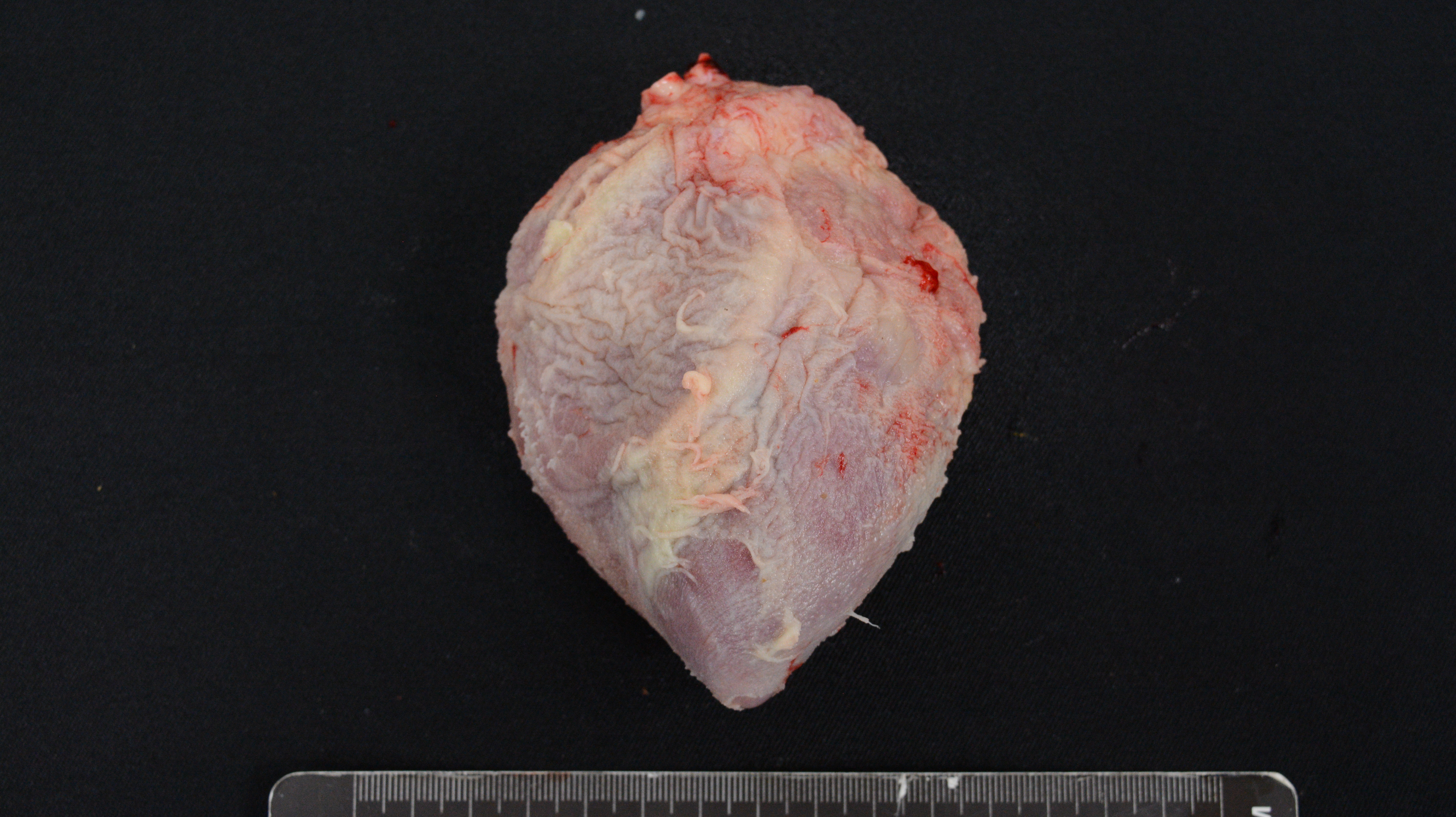

Supplement: Supplemental Information 4 [file peerj-06-6017-s004.jpg]

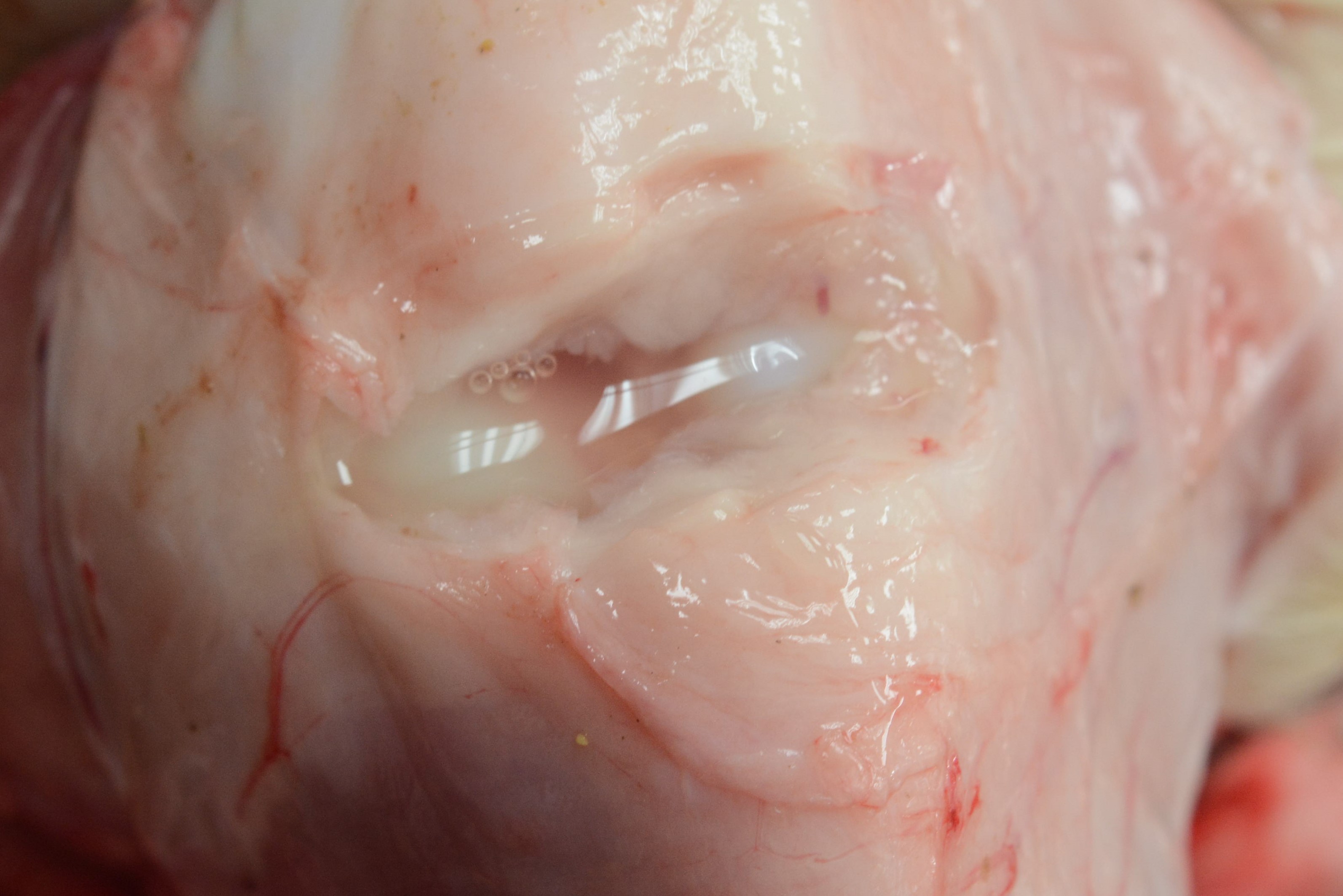

Supplement: Supplemental Information 5 [file peerj-06-6017-s005.jpg]

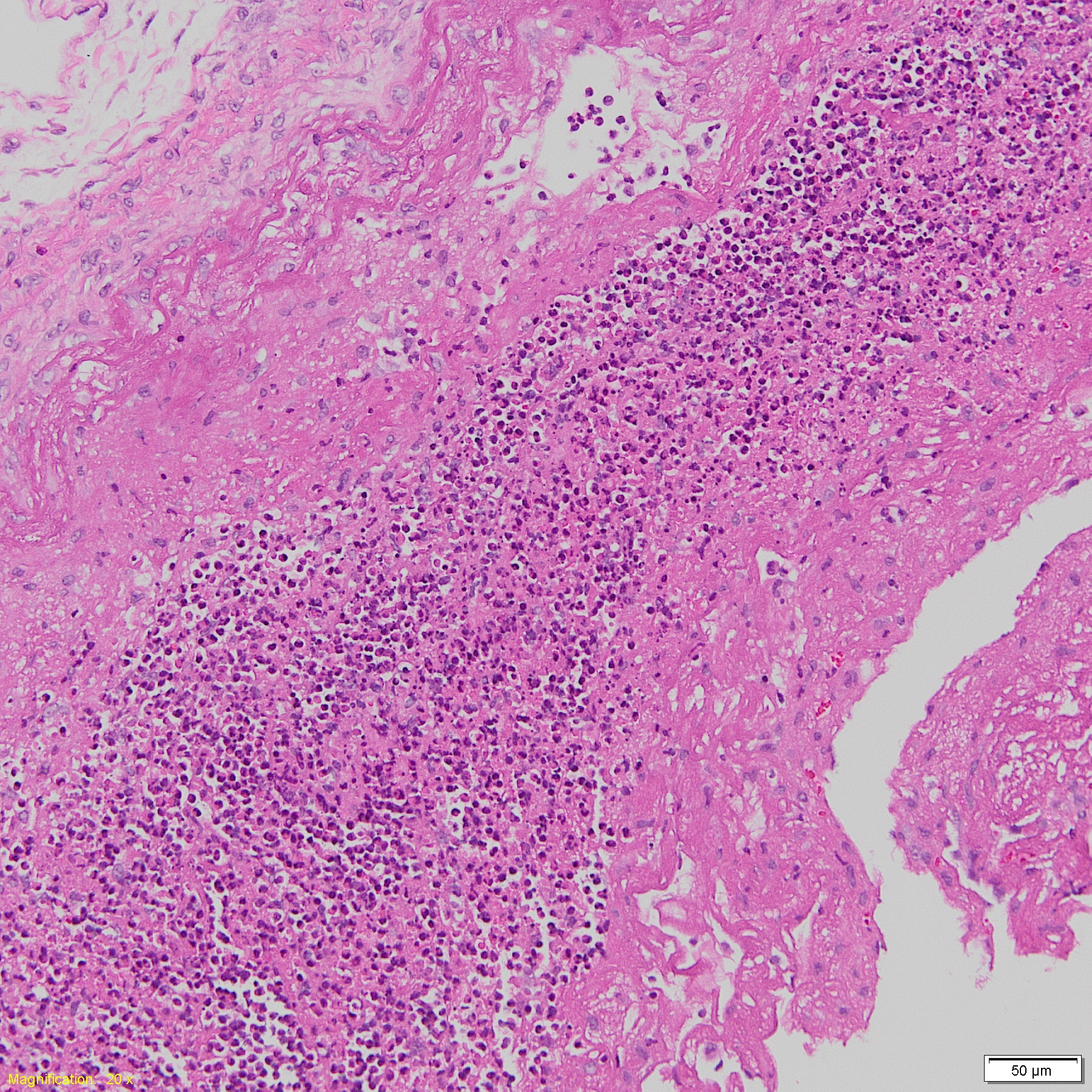

Supplement: Supplemental Information 6 [file peerj-06-6017-s006.jpg]

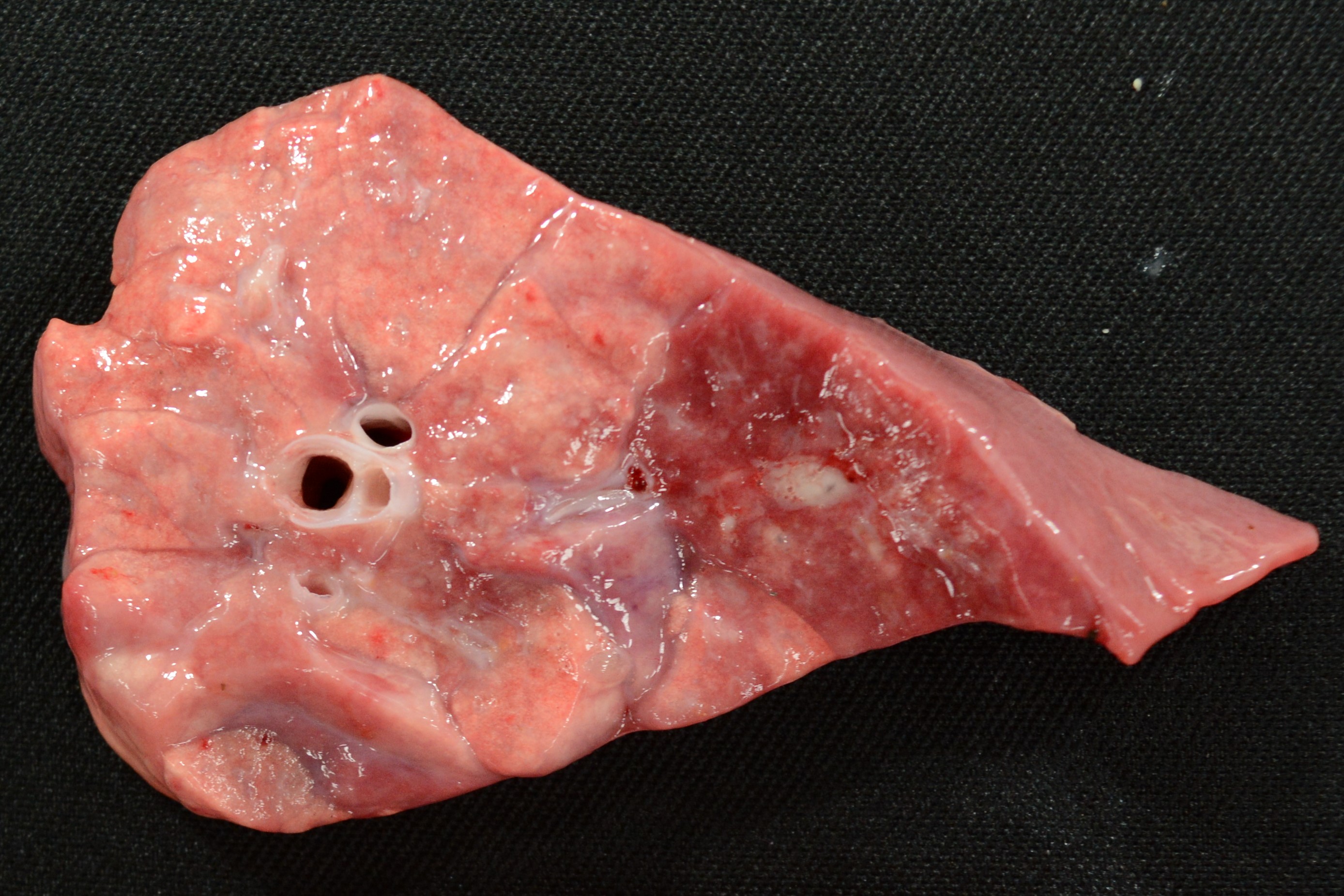

Supplement: Supplemental Information 7 [file peerj-06-6017-s007.jpg]

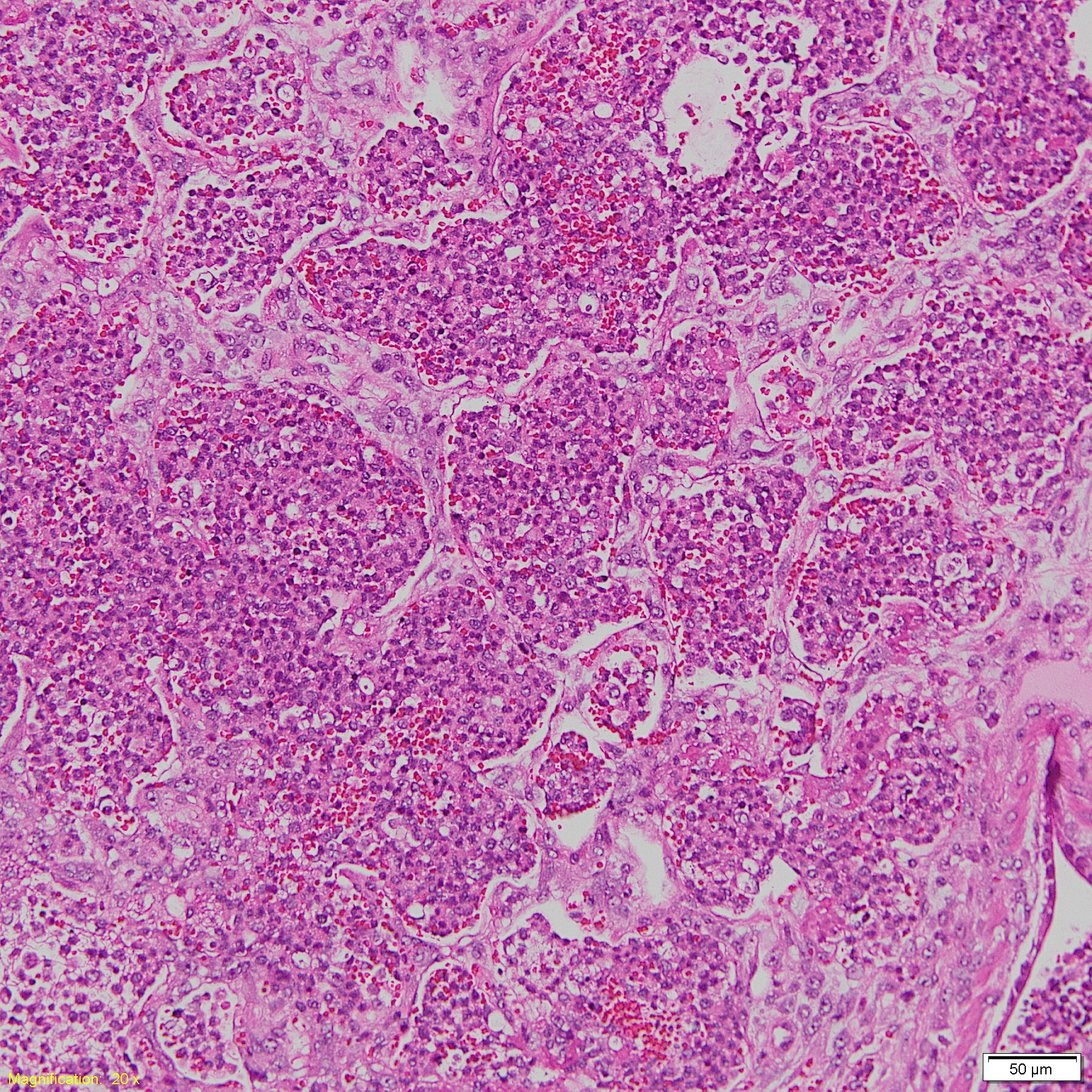

Supplement: Supplemental Information 8 [file peerj-06-6017-s008.jpg]

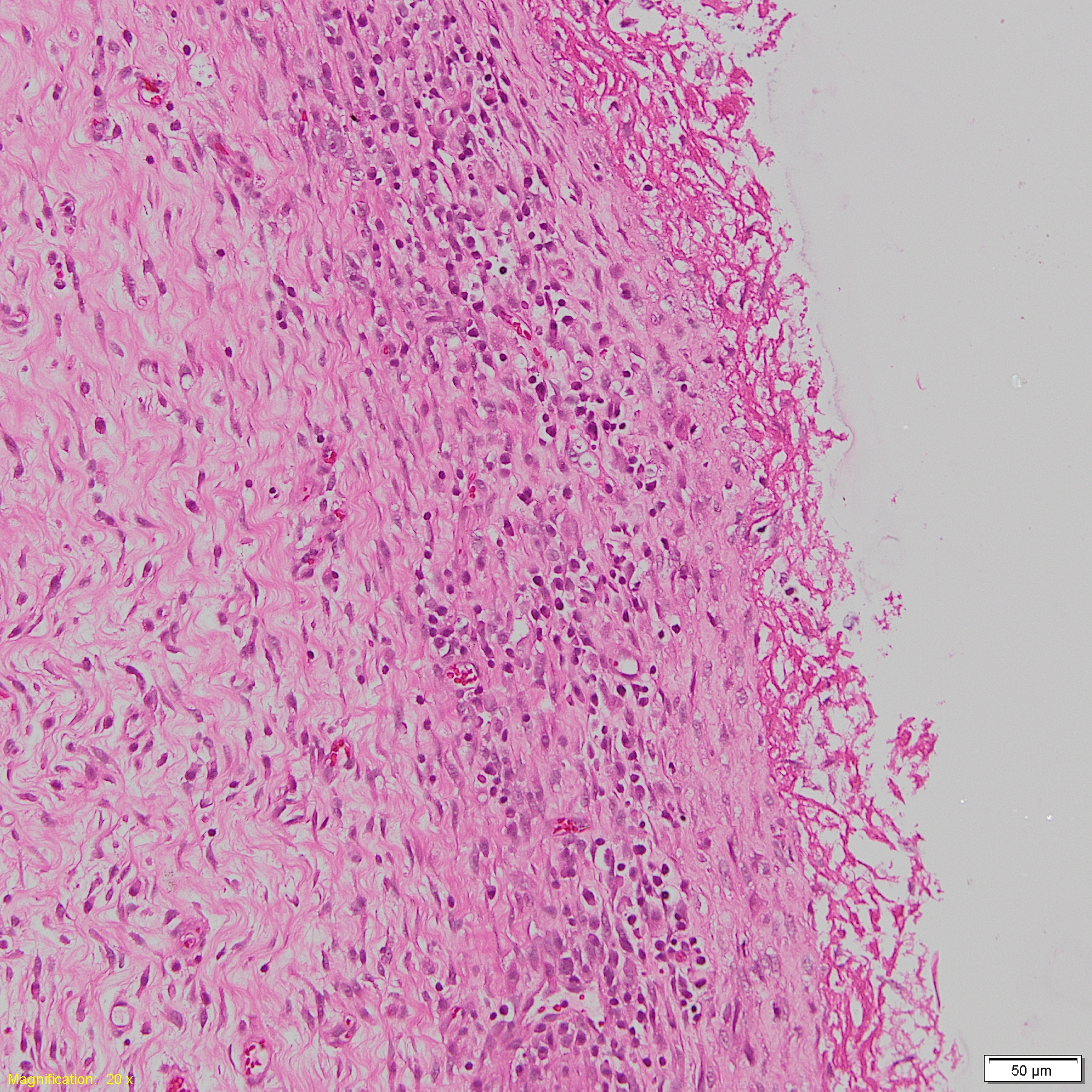

Supplement: Supplemental Information 9 [file peerj-06-6017-s009.jpg]

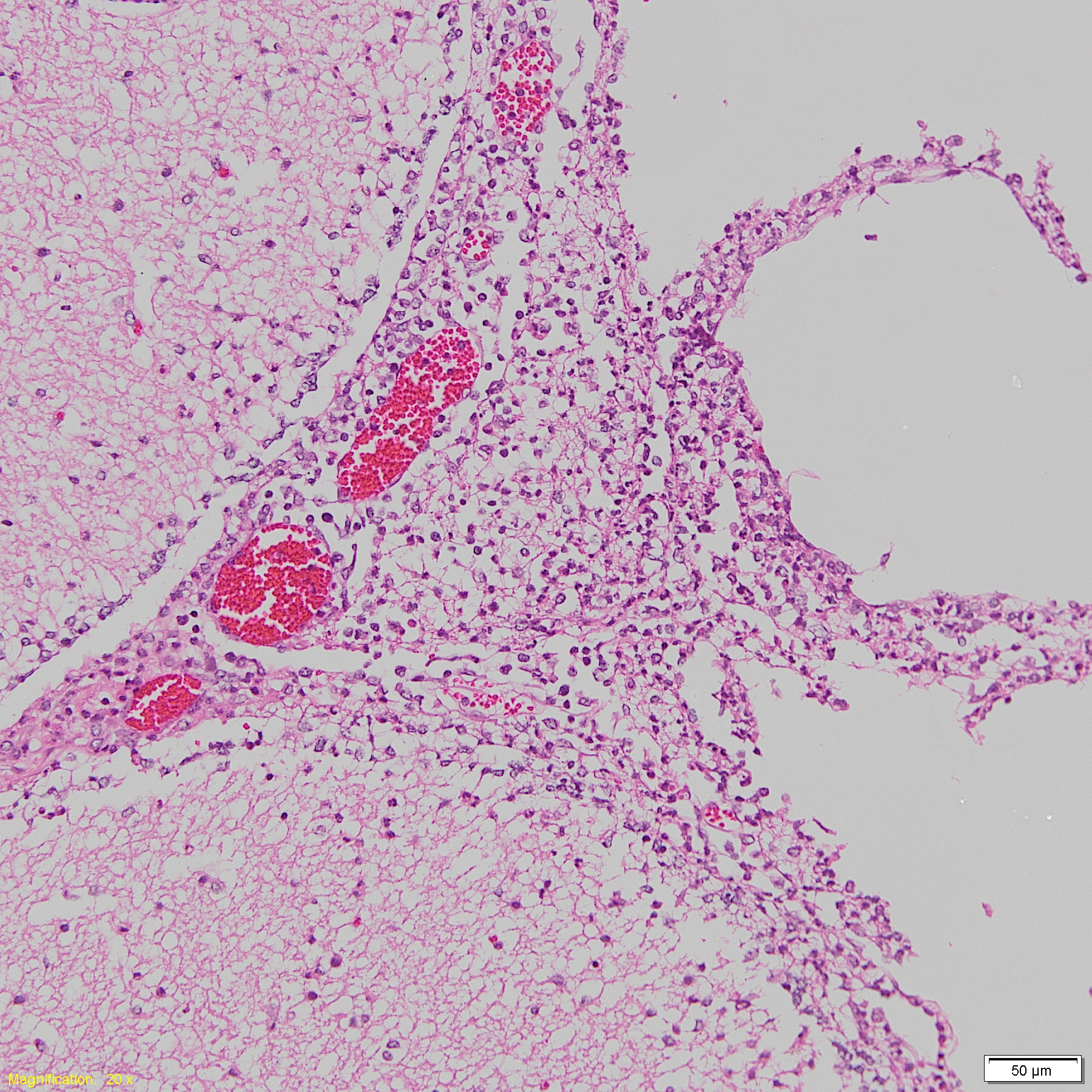

Supplement: Supplemental Information 10 [file peerj-06-6017-s010.jpg]

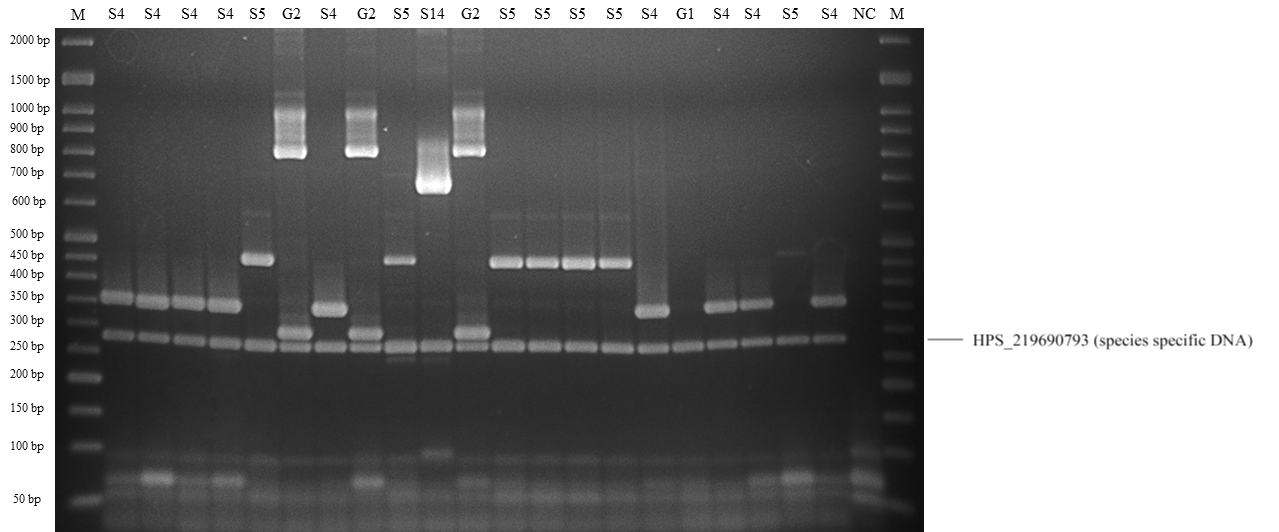

Supplement: Supplemental Information 11 — Lane M: 50 bp DNA Ladder, lane S5: serovar 5 or 12, lane G2: molecular serotyping non-typable group 2, lane S4: serovar 4, lane S9: serovar 9, lane G1: molecular serotyping non-typable group 1, lane NC: negative control. [file peerj-06-6017-s011.png]

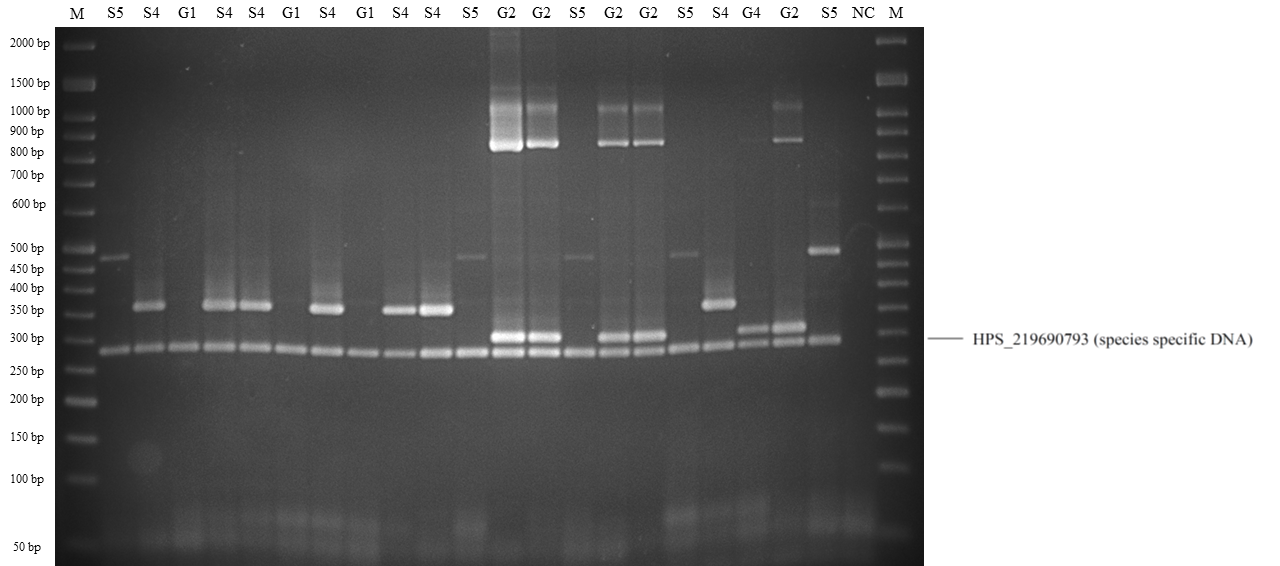

Supplement: Supplemental Information 12 — Lane M: 50 bp DNA Ladder, lane S4: serovar 4, lane S5: serovar 5 or 12, lane G2: molecular serotyping non-typable group 2, lane S14: serovar 14, lane G1: molecular serotyping non-typable group 1, lane NC: negative control. [file peerj-06-6017-s012.png]

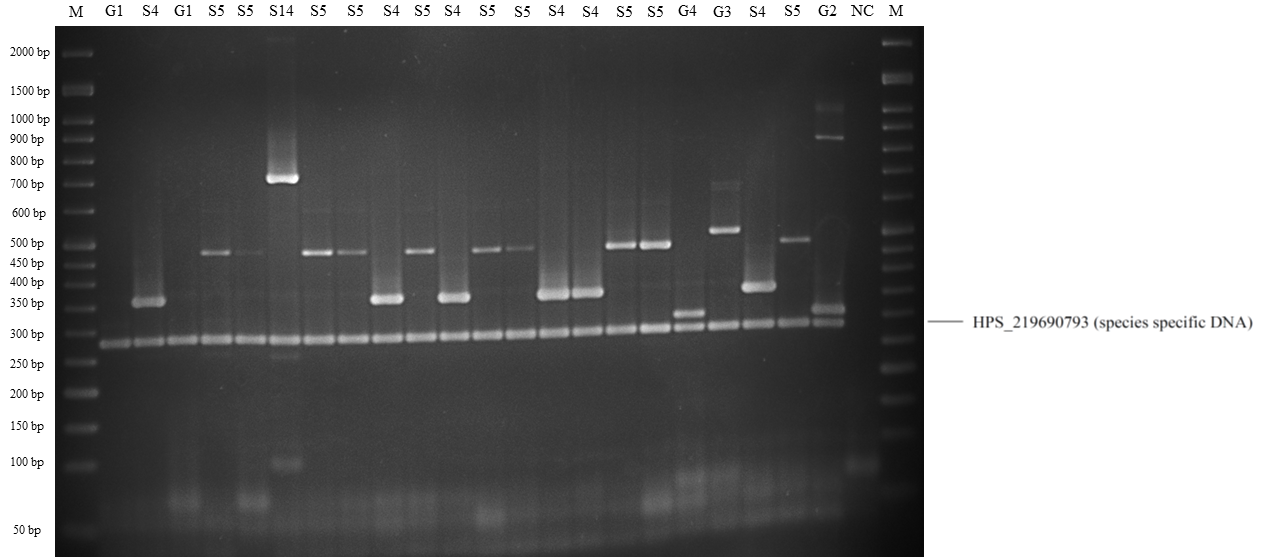

Supplement: Supplemental Information 13 — Lane M: 50 bp DNA Ladder, lane S5: serovar 5 or 12, lane S4: serovar 4, lane G1: molecular serotyping non-typable group 1, lane G2: molecular serotyping non-typable group 2, lane NC: negative control. Histopathological bronchopneumonia lesion in H. parasuis infected pigs. [file peerj-06-6017-s013.png]

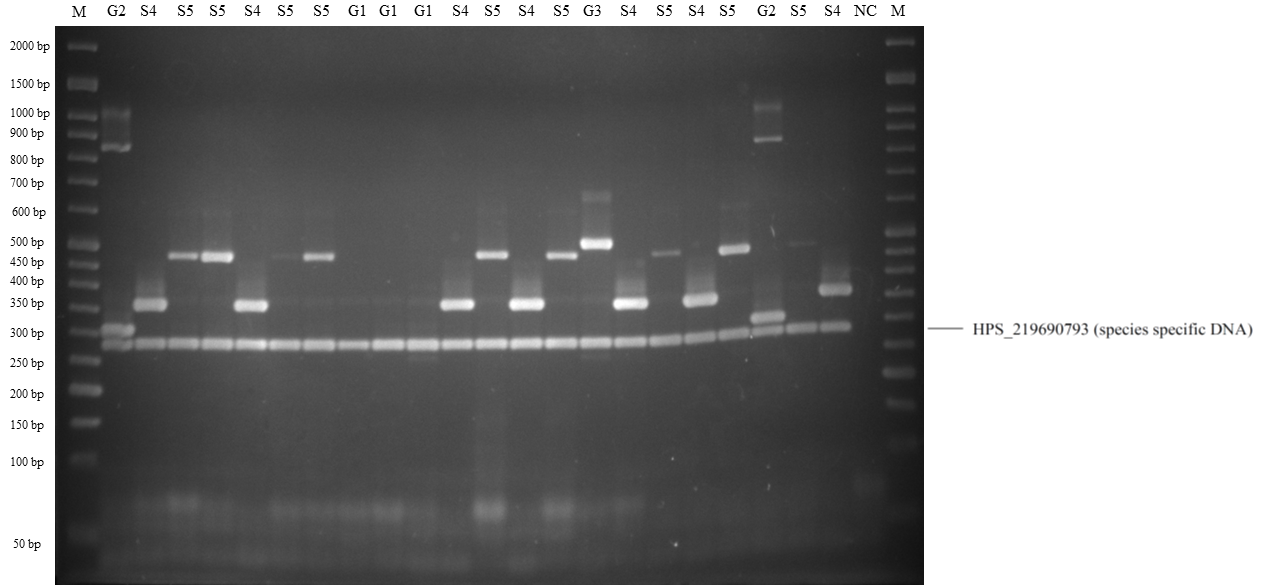

Supplement: Supplemental Information 14 — Lane M: 50 bp DNA Ladder, lane G1: molecular serotyping non-typable group 1, lane S4: 608 serovar 4, lane S5: serovar 5 or 12, lane S14: serovar 14, lane G4: molecular serotyping non- 609 typable group 4, lane G3: molecular serotyping non-typable group 3, lane NC: negative control. [file peerj-06-6017-s014.png]

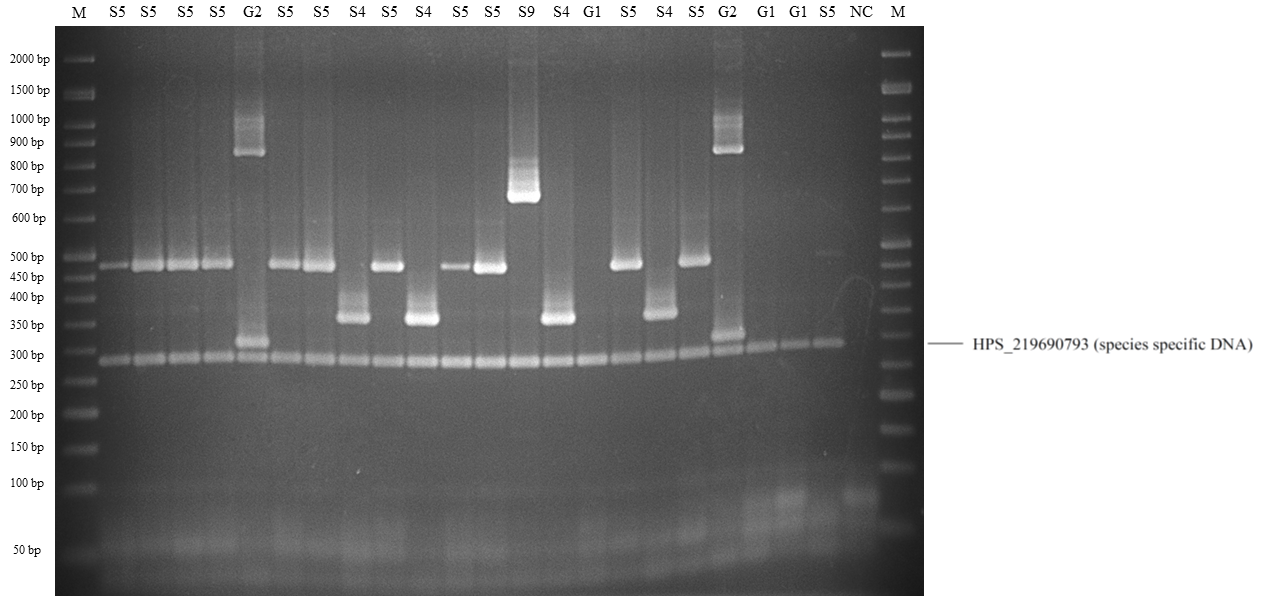

Supplement: Supplemental Information 15 — Lane M: 50 bp DNA Ladder RTU (GeneDireX), lane G2: molecular serotyping non-typable group 2, lane S4: serovar 4, lane S5: serovar 5 or 12, lane G1: molecular serotyping non-typable group 1, lane G3: molecular serotyping the non-typable group 3, lane NC: negative control. [file peerj-06-6017-s015.png]
