# Supplementary material for: Molecular serotyping of Haemophilus parasuis isolated from diseased pigs and the relationship between serovars and pathological patterns in Taiwan
Source: PeerJ. 2018 Nov 29;6:e6017. doi: 10.7717/peerj.6017 (PMC6275120; doi:10.7717/peerj.6017)
Supplement: Supplemental Information 16 [file peerj-06-6017-s016.docx]

Supplementary Table 1 Serovar distribution of *Haemophilus parasuis* in different countries or regions by different serotyping assays

| Continent | Country | Published  date | Sampling  time | Isolate | Method† | The most prevalent serovars | | | NT‡ | Reference |
| --- | --- | --- | --- | --- | --- | --- | --- | --- | --- | --- |
|  |  |  |  |  |  | First | Second | Third |  |  |
| Europe | Germany | 1992 | 5 years | 290 | GID | S5  (23.8%) | S4  (17.2%) | S2  (5.5%) | 26.2% | (Kielstein & Rapp-Gabrielson 1992) |
|  | Spain | 1999 | 1993 to 1997 | 327 | GID | S5  (18.4%) | S4  (16%) | S2  (9.2%) | 29.3% | (Rubies et al. 1999) |
|  | Spain | 2003 | 1998 to 2002 | 67 | GID | S5  (17.9%) | S4  (16.4%) | S7  (6%) | 37.3% | (Del Rio et al. 2003) |
|  |  |  |  |  | IHA | S5  (22.4%) | S4  (19.4%) | S7  (10.4%) | 7.5% |  |
|  | Denmark | 2004 | 1998 to 2002 | 103 | IHA | S5  (36%) | S4  (13%) | S13  (22%) | 15.0% | (Angen et al. 2004) |
|  | The Netherlands | 2012 | 2007 to 2009 | 117 | IHA | S4  (23.1%) | S2 or S13  (14.5%) | S1  (11.1%) | 13.7% | (Dijkman et al. 2012) |
|  | Italy | 2013 | 2007 to 2011 | 106 | GID | S4  (24.5%) | S13  (19.8%) | S5  (11.3%) | 27.3% | (Luppi et al. 2013) |
|  | The United Kingdom | 2015 | 2013 to 2014 | 84 | IHA§ | S4  (22.6%) | S5/12  (13.1%) | S1 or S14  (4.8%) | 17.9% | (Howell et al. 2015) |
|  |  |  |  |  | mPCR | S4  (29.8%) | S5/12  (22.6%) | S7, S13 or S15  (8.3%) | 0% |  |
| America | The United States  and Canada | 1992 | Non-mentioned | 243 | GID | S5  (24.3%) | S4  (16.1%) | S2  (8.2%) | 15.2% | (Rapp-Gabrielson & Gabrielson 1992) |
|  | North American | 2003 | 1999 to 2001 | 98 | GID | S4  (39%) | S3  (8%) | S1 or S12  (7%) | 27.0% | (Oliveira et al. 2003) |
|  | Canada | 2004 | 1991 to 2002 | 250 | GID | S4  (27%) | S5  (15%) | S13  (14%) | >30% | (Tadjine et al. 2004) |
|  | The United States | 2004 | 1991 to 2002 | 50 | GID | S4  (25%) | S12  (23%) | S5  (15%) | >30% | (Tadjine et al. 2004) |
|  | Brazil | 2012 | Non-mentioned | 40 | GID | S4  (26.1%) | S5  (17.4%) | S14  (8.7%) | 39.0% | (Castilla et al. 2012) |
| Australia | Australia | 1996 | Non-mentioned | 31 | GID | S5  (22.6%) | S13  (19.4%) | S4  (12.9%) | 16.1% | (Blackall et al. 1996) |
| Asia | China | 2005 | 2002 to 2004 | 281 | GID | S4  (19.2%) | S5  (13.5%) | S13  (10.3%) | 36.7% | (Cai et al. 2005) |
|  |  |  |  |  | IHA | S4  (25.3%) | S5  (21%) | S13  (12.1%) | 9.6% |  |
|  | China | 2016 | 2007 to 2014 | 100 | GID | S5/12  (38%) | S4  (15%) | S7  (7%) | 27.0% | (Ma et al. 2016) |
|  |  |  |  |  | mPCR | S5/12  (40%) | S4  (33%) | S7  (6%) | 7.0% |  |
|  | Taiwan | This study | 2013 to 2017 | 132 | mPCR | S5/12  (37.6%) | S4  (27.8%) | S14  (2.3%) | 30.1% | This study |
|  |  |  |  |  | mPCR  with sequence | S5/12  (37.6%) | S4  (27.8%) | S13  (15%) | 13.5% |  |

†Gel immuno-diffusion test (GID); indirect hemagglutination test (IHA); mPCR (molecular serotyping polymerase chain reaction)

‡Non-typable including conventional serotyping cross-reactive and non-typable, and molecular serotyping non-typable

§Cross-reactions ignored
