# Supplementary material for: Molecular serotyping of Haemophilus parasuis isolated from diseased pigs and the relationship between serovars and pathological patterns in Taiwan
Source: PeerJ. 2018 Nov 29;6:e6017. doi: 10.7717/peerj.6017 (PMC6275120; doi:10.7717/peerj.6017)
Supplement: Supplemental Information 19 [file peerj-06-6017-s019.docx]

Supplemental Table 4 Relationship between pathological diagnoses and serovars

| Serovar | Pathological lesion pattern | | | |
| --- | --- | --- | --- | --- |
|  | Serositis with respiratory | Serositis | Respiratory | Total |
| 2 | 1 (100%) | 0 (0%) | 0 (0%) | 1 |
| 4 | 12 (57.1%) | 8 (38.1%) | 1 (4.8%) | 21 |
| 5 or 12 | 26 (24.1%) | 17 (37.8%) | 2 (4.4%) | 45 |
| 7 | 0 (0%) | 1 (100%) | 0 (0%) | 1 |
| 9 | 1 (100%) | 0 (0%) | 0 (0%) | 1 |
| 13 | 9 (42.9%) | 12 (57.1%) | 0 (0%) | 21 |
| 14 | 1 (50%) | 1 (50%) | 0 (0%) | 2 |
| MSNT group 1 | 9 (69.2%) | 3 (23.1%) | 1 (7.7%) | 13 |
| 4 and 7† | 0 (0%) | 1 (100%) | 0 (0%) | 1 |
| 4 and MSNT group 1† | 0 (0%) | 1 (100%) | 0 (0%) | 1 |
| 5 and 13† | 0 (0%) | 1 (100%) | 0 (0%) | 1 |
| Total | 59 (54.6%) | 45 (41.7%) | 4 (3.7%) | 108 |

†co-infection two serovars in one case
